# Audit of mammography requests in Abakaliki, South-East Nigeria

**DOI:** 10.1186/s12957-017-1122-7

**Published:** 2017-03-07

**Authors:** UE Eni, KC Ekwedigwe, I Sunday-Adeoye, ABC Daniyan, ME Isikhuemen

**Affiliations:** 1 Department of Surgery, Federal Teaching Hospital, Abakaliki, Nigeria; 2 National Obstetric Fistula Centre, Abakaliki, Nigeria

**Keywords:** Breast cancer screening, Screening mammography, Diagnostic mammography, Audit, Abakaliki, Nigeria

## Abstract

**Background:**

Breast cancer is the leading cancer in women in both developed and developing countries. Screening mammography detects breast cancer even before a lump can be palpated, with better prognosis. The introduction of mammographic technique for screening breast cancer, despite its importance, has been slow to adopt and virtually non-existent in many parts of Sub-Saharan Africa including Nigeria. For this reason, the indications of mammography have not been well defined in our setting. The aim of this study was to audit our mammography requests, with a view to improving its application in our setting.

**Methods:**

This is a descriptive study carried out on 69 female patients who had mammography at the National Obstetric Fistula Centre, Abakaliki, from January 2014 to December 2015. Findings on clinical examination were entered in a proforma. Mammography was performed in craniocaudal and mediolateral views using the Lorad M-IV (film-screen) mammography machine. Data was analysed using the Statistical Package for Social Sciences (SPSS) version 21.

**Results:**

All 69 patients were females. Their mean age was 42.1 ± 11 years. Majority of the patients (69.6%) were between 30 and 49 years. The commonest indication for mammography was breast lump which was found in 46 patients (66.7%). Breast pain was present in 36 (52.2%) of patients. The different Breast Imaging Reporting and Data System (BIRADS) categories were BIRADS 0: 20 (28.99%), BIRADS 1: 8 (11.59%), BIRADS 2: 9 (13.04%), BIRADS 3: 4 (5.8%), BIRADS 4: 19 (27.54%) and BIRADS 5: 9 (13.04%).

**Conclusions:**

Diagnostic mammography remains the commonest indication for mammography in our setting. Public awareness, poverty reduction and ready availability of mammography facilities are required to improve screening mammography in our setting.

## Background

Breast cancer is the leading cancer among women both in developed and developing countries [1]. In 2015, an estimated 231,840 new cases of invasive breast cancer was expected to be diagnosed among women, as well as an estimated 60,290 additional cases of in situ breast cancer [2], mostly from screening investigations in developed countries. In 2015, approximately 40,290 women were estimated to die from breast cancer [2].

In Nigeria, most cases present at advanced stages with minimal hope of any intervention that will reasonably reduce disability and mortality [3]. The reasons for late presentation among African women with breast cancer include negative symptom interpretation, fear of the unknown, belief in alternative medicine, ignorance, poverty, lack of trust and confidence in orthodox medicine and limited access to appropriate healthcare [4]. Various breast cancer awareness advocacy groups are helping to address the issue of ignorance and late presentation. Adoption of triple assessment helps improve diagnostic accuracy of breast lesions especially in the early stages. Triple assessment of breast diseases includes imaging, clinical assessment and histology [5, 6].

So far, the only breast cancer screening method that has proved to be effective is mammography screening among women that are qualified. [2, 6]. Screening mammography detects breast cancer before a lump can be felt, with better prognosis [6]. The introduction of mammographic technique for screening breast cancer, despite its importance, has been slow in many parts of Sub-Saharan Africa, including Nigeria [7]. Sensitivity ranging from 76.0 - 92.0% and specificity ranging from 74.8-96.2% have been reported for mammography [8]. Indications for mammography are screening and diagnostic. The American College of Radiology (ACR) has developed the Breast Imaging Reporting and Data System (BIRADS), which is intended to standardize the terminology in mammographic reports [9].

The aim of this study was to audit mammography requests in the study facility, with a view to improving its application in our setting.

## Methods

This was a 2-year descriptive study carried out on 69 female patients who had mammography at the National Obstetric Fistula Centre, Abakaliki, from January 2014 to December 2015. Apart from providing free services to patients with genitourinary fistula, the Centre has a general surgery clinic dedicated for the screening, diagnosis and management of breast diseases. All patients with breast symptoms were evaluated clinically, and relevant investigations including mammography when indicated were done. Mammography was performed in craniocaudal and mediolateral views using the Lorad M-IV (film-screen) mammography machine. A consultant radiologist reported the mammograms according to the American College of Radiologist-Breast Imaging Reporting and Data System (ACR-BIRADS) classification. Fine needle aspiration cytology (FNAC) was done for patients that had significant breast mass, followed by incision or excision biopsy when necessary. Findings on clinical examination, mammography and FNAC were documented. Data were analysed using SPSS version 21. For the purpose of this study, BIRADS categories 1, 2 and 3 were classified as negative while BIRADS categories 4 and 5 were classified as positive results. BIRADS 0 was inconclusive. Ethical clearance was gotten from the institutions’ ethical committee. This was also in line with ethical standards laid down in the declaration of Helsinki. Appropriate treatment was offered to each patient following diagnosis.

## Results

Their mean age was 42.1 ± 11 years. The age range was between 18 and 68 years. Most of the patients were between 30 and 49 years (69.6%). This is shown in table 1

**Table 1:** Age distribution of patients

Age	Frequency (%)
10–19	1 (1.4)
20–29	4 (5.8)
30–39	26 (37.7)
40–49	22 (31.9)
50–59	10 (14.5)
60–69	6 (8.7)
Total	69 (100)

The commonest indication for mammography was breast lump which was found in 46 patients (66.7%). Breast pain was present in 52.2% (Table 2).

**Table 2:** Presenting complaint of patients for which mammography was done

	Frequency (%)
Lump	46 (66.7)
Pain	36 (52.2)
Nipple discharge	8 (11.6)
Ulceration	1 (1.4)

The different BIRADS categories (Table 3) were BIRADS 0: 20 (28.99%), BIRADS 1: 8 (11.59%), BIRADS 2: 9 (13.04%), BIRADS 3: 4 (5.8%), BIRADS 4: 19 (27.54%) and BIRADS 5: 9 (13.04%).

**Table 3:** Mammography findings according to BIRADS category

Category	Frequency (%)
0	20 (28.99)
1	8 (11.59)
2	9 (13.04)
3	4 (5.8)
4	19 (27.54)
5	9 (13.04)
Total	69 (100)

A total of 28 (40.6%) had positive BIRADS and a total of 21 (30.4%) had negative BIRADS as shown in Table 4. BIRADS 1, 2 and 3 were classified as negative BIRADS while BIRADS 4 and 5 were classified as positive BIRADS.

**Table 4:** Positive and negative BIRADS (*n* = 69)

BIRADS category	Frequency (%)
Positive	28 (40.6)
Negative	21 (30.4)
Inconclusive	20 (29)
Total	69 (100)

## Discussion

There are many advanced imaging modalities for breast evaluation today. However, the only breast cancer screening method that has proved to be effective is mammography. The most important advantage of mammography is detecting small cancers even before a lump can be felt which offers a better prognosis [6].

In this study, negative test results (BIRADS 1, 2 and 3) were detected in 40.6%, while positive test results (BIRADS 4 and 5) were detected in 30.4% of respondents. This is at variance with a study done in Iran where up to 91.3% of respondents had negative test results [10]. Proportion of patients with positive BIRADS was quite high in this study compared to those with negative BIRADS because most of our respondents had breast complaint and had diagnostic mammography rather than screening mammography. In this study, 11.59% of respondents had BIRADS category 1. This is similar to a study done in Lagos, Nigeria, where 13.7% of respondents had BIRADS category 1 [11]. This similarity may have been due to the fact that a significant number of women in that study had mastalgia which was also present in our study. This is at variance with a study done in Osun State, Nigeria, where BIRADS category 1 was 77.37% [7]. The difference in the results may be explained by the fact that the study done in Osun State was on screening mammography.

In a study done in Benin City, Nigeria, among health workers, only 3.1% of respondents greater than 40 years have done mammography [12]. This study which was done among health workers indicated a poor uptake of mammography. Screening mammography is still largely underutilized in our environment. The reason is attributable to ignorance and paucity of screening mammography facilities [7]. The mammography machine at the National Obstetric Fistula Centre, Abakaliki, where this study was done is the only functional one in Ebonyi State presently and serves the state as well as nearby towns of neighbouring states of Abia, Cross River, Enugu and Benue States. Most of our requests were for diagnostic mammography, to verify suspicious breast symptoms. Fourty-six (66.7%) of the study population had breast lump, 52.2% had breast pain, 11.6% had nipple discharge while 1.4% had skin ulceration.

## Conclusions

Mammography requests in the study environment are mainly for diagnostic purposes. Screening mammography is yet to be fully utilized. Mammography should be strongly advocated as part of the triple assessment for breast diseases. We recommend mammography as a method for both screening and evaluation of breast diseases. Public awareness, poverty reduction and ready availability of mammography facilities are required to improve screening mammography in our setting. A subsidized national screening programme may aid in the utilization of screening mammography.

## Abbreviations

ACR: American College of Radiologist
BIRADS: Breast Imaging Reporting and Data System
FNAC: Fine Needle Aspiration Cytology

## References

[1] Parkin DM, Bray F, Ferlay J, Pisani P (2005). Global cancer statistics. CA Cancer J Clin.

[2] American Cancer Society (2015). Breast cancer facts & figures 2015–2016.

[3] Okobia MN, Bunker CH, Okonofua FE, Osime U (2006). Knowledge attitude and practice of Nigerian women towards breast cancer: a cross-sectional study. World J Surg Oncology.

[4] Donkor A (2015). Factors contributing to late presentation of breast cancer in Africa: a systematic review. Arch Med.

[5] Ifeanyichukwu AO (2015). Assessment of breast cancer screening practices among women of reproductive age in Benin City, Edo State. Int J Trop Dis Health.

[6] American Cancer Society. Breast cancer facts & figures 2005–2006. Atlanta: American Cancer Society, Inc.; 2005.

[7] Bello TO, Ojemakinde MO, Aremu AA, Ojemakinde KO, Agodirin SO. Screening mammography features in Nigerian women: a pilot study. Afr J Med Sci. 2012;41.

[8] Debono JC, Poulos AE, Houssami N, Turner RM, Boyages J. Evaluation of radiographers’ mammography screen-reading accuracy in Australia. J Med Radiat Sci. 2015; 62(1):15–22. 10.1002/jmrs.59 PMC4364802 26229663

[9] American College of Radiology (2013). Breast imaging reporting and data system (BI-RADS).

[10] Ehsanbakhsh AR, Toosi FS, Khorashadizadeh N (2009). Different BIRADS categories in screening and diagnostic mammography. Iran J Radiol.

[11] Akinola RA, Akinola OI, Shittu LAJ, Balogun BO, Tayo AO (2007). Appraisal of mammography in Nigerian women in a new teaching hospital. Sci Res Essay.

[12] Akhigbe AO, Omuemu VO (2009). Knowledge, attitudes and practice of breast cancer screening among female health workers in a Nigerian urban city. BMC Cancer.

